# Multidrug Resistant Brain Abscess Due to *Acinetobacter baumannii* Ventriculitis Cleared by Intraventricular and Intravenous Tigecycline Therapy: A Case Report and Review of Literature

**DOI:** 10.3389/fneur.2018.00518

**Published:** 2018-06-29

**Authors:** Wenyong Long, Jian Yuan, Jingping Liu, Jinfang Liu, Ming Wu, Xin Chen, Gang Peng, Changwu Wu, Chi Zhang, Xiangyu Wang, Wei Zhao, Qing Liu

**Affiliations:** ^1^Department of Neurosurgery, Xiangya Hospital, Central South University, Changsha, China; ^2^Zhongshan School of Medicine, Sun Yat-sen University, Guangzhou, China

**Keywords:** intracranial infection, multidrug-resistant, tigecycline therapy, *Acinetobacter baumannii*, ventriculitis

## Abstract

**Objective:** Ventricular infection from multidrug-resistant (MDR) *Acinetobacter baumannii* (*A. baumannii*) is one of the most severe complications of craniotomy. However, the availability of effective therapeutic options for these infections is limited. Thus, this report aims to describe the efficacy of abscess clearance by intraventricular and intravenous tigecycline therapy in managing patients with multidrug-resistant *A. baumannii* ventriculitis after neurosurgery. Moreover, the current literature on the use of tigecycline therapy for these life-threatening infections is reviewed and summarized, and a treatment regimen based on the available data is proposed.

**Methods:** A patient with multidrug-resistant *A. baumannii* ventriculitis was admitted in our hospital and was provided with a detailed therapeutic schedule. Tigecycline treatments for multidrug-resistant *A. baumannii* ventriculitis that were reported in the literature were also reviewed and summarized.

**Results:** The patient in our hospital underwent abscess clearance on a ventriculoscope and was subsequently subjected to multi-route tigecycline therapy 14 days after the start of the continuous ventricular irrigation (CVI) tigecycline and 3 days after the intraventricular (IVT) tigecycline. The signs of ventriculitis disappeared, and the Acinetobacter cerebrospinal fluid (CSF) load steadily decreased until CSF sterilization. Literature review identified seven cases of ventricular infection from multidrug-resistant *A. baumannii* treated with tigecycline. In the eight cases, all patients were male adults (>18 years), with a mean age of 46.1 (range: 22–75) years. Meningitis/ventriculitis was secondary to neurosurgery procedures for the management of various central nervous system diseases in all cases. A good clinical outcome was achieved in all eight patients with multidrug-resistant *A. baumannii* meningitis/ventriculitis treated with CVI and/or IVT tigecycline, and any relevant complications were not observed.

**Conclusions**: CVI and IVT tigecycline and IVT colistin could be considered as the first-line therapy in patients with ventricular infections from MDR/extreme drug-resistant *A. baumannii*. However, more studies should be conducted to confirm our observation.

## Introduction

Ventricular infection from multidrug-resistant (MDR) *Acinetobacter baumannii* (*A. baumannii*) is one of the most severe complications of craniotomy, with a mortality rate reaching up to 71% (1), and the drugs used to treat these infections have poor CSF penetration. Intraventricular (IVT) and intravenous (IV) colistin are used as the last resort of treatment of MDR *A. baumannii* ventriculitis/meningitis. However, the colistin-associated neurotoxicity, reported in up to 21.7% of the cases (2), limits its use. Tigecycline is an antibiotic with an excellent activity against a broad spectrum of MDR pathogens, including *Acinetobacter baumannii* and *Klebsiella pneumoniae*, with a favorable toxicity profile. The use of tigecycline received approval from the Food and Drug Administration in the USA in 2005, and currently, this drug can only be administered intravenously (3). The effectiveness of tigecycline for treating intracranial *A. baumannii* infections is still controversial because of its low CSF penetration level (4). Thus, the treatment with continuous ventricular irrigation (CVI) and IVT tigecycline may be a fusible way forward.

Herein, we describe the first case of ventriculitis and abscess by MDR *A. baumannii* and clearance following CVI and IVT tigecycline therapy.

Previous case reports and case series, which provided information on the efficacy or safety of tigecycline for ventriculitis or meningitis based on a positive CSF culture (with MDR or XDR *A. baumannii*), were included. Moreover, ventriculitis or meningitis in this study should meet the following criteria: positive CSF culture, fever >38°C, increased white blood cells (>10 cells/mm3 with >50% polymorph), and increased protein and/or decreased glucose levels in the CSF (5).

The reviewed six articles which met the inclusion criteria, and a total of seven patients with meningitis/ventriculitis due to MDR *A. baumannii*, were included and treated with tigecycline (6–10, 11). Moreover, we included one case of *A. baumannii* (AB) ventriculitis, with antibiograms for the isolates of AB only susceptible to tigecycline, which were treated with abscess clearance on a ventriculoscope combined with CVI and IVT tigecycline treatment. We followed up the results of the laboratory CSF analusis and the cultures of the specimens to assess the microbiological clearance of the infecting organisms (Tables 1, 2).

**Table 1:** Studies regarding (IV or CVI or IVT) administration of tigecycline in *Acinetobacter baumannii* meningitis/ventriculitis (I).

**Reference**	**(10)**	**(11)**	**(11)**	**This study**
Age	25	48	52	55
Sex	male	male	male	male
Underlying disease(s)	Pilocytic astrocytom hydrocephalus	Vertebral trauma	Lumbar disk herniation	Intracerebellar hemorrhage, CSF, hydrocephalus
Foreign body	EVD	Spinal instrumentation	None	EVD
Days from admission to diagnosis	11	10	9	26
Antimicrobial susceptibilities AB Colimycin;	Susceptible to TGC (MIC = 3.2 μg/mL); MDR	Susceptible to netilmicin, TGC(MIC = 0.38 μg/mL); MDR	Susceptible to netilmicin, TGC(MIC = 0.38 μg/mL); MDR	Susceptible to TGC(MIC = 16 μg/mL); XDR
Current antimicrobial regimens	TGC and colimycin and meropenem	TGC, Netilmicin, and meropenem	TGC, Netilmicin	TGC, cefoperazone-sulbactam, amikacin
IV/CVI/IVT, tigecycline	IV, 50 mg/q12h	IV, 50 mg/q12h	IV, 50 mg/q12	IV, 100 mg/q12h CVI, 10 mg/q12h IVT, 2 mg/q12h
Co-administered antibiotics	Colimycin IV, 9MIU/q24	Netilmicin IV, (400 mg/q24h) Meropenem IV, (2g/q8h)	Netilmicin IV, (400 mg/q24h) Meropenem IV, (2g/q8h)	Cefoperazone-sulbactam IV, (2g/q8h)
Days to CSF sterilization	23	21	21	12
Toxicity	None	None	None	None
Infection outcome	Cured	Cured	Cured	Cured
Survival	Yes	Yes	Yes	Yes

*CSF, cerebrospinal fluid; MIC, minimum inhibitory concentration; EVD, external ventricular drain; MDR, multidrug resistant; XDR, extensively drug-resistant; TGC, tigecycline; q8h, every 8 h; q12h, every 12 h; q24h, every 24 h; IV, intravenous; CVI, continuous ventricular irrigation; IVT, intraventricular*.

**Table 2:** Studies regarding (IV or CVI or IVT) administration of tigecycline in *Acinetobacter baumannii* meningitis/ventriculitis (II).

**Reference**	**(6)**	**(7)**	**(8)**	**(9)**
Age	75	22	50	42
Sex	male	male	male	male
Underlying disease(s)	Frontal contusion, subdural hematoma	Giant pituitary adenoma, CSF leak	Cranial traumatic brain injury hydrocephalus	Ependymoma 4th ventricle, CSF leak
Foreign body	EVD	Fibrin glue, dural substitutes	No	EVD
Days from admission to diagnosis	4	18^a^	31	15
Antimicrobial susceptibilities	Susceptible to TGC, CST; XDR	Susceptible to TGC, (MIC = 2 μg/mL); MDR	Susceptible to TGC; XDR	Susceptible to TGC(MIC = 0.5 μg/mL) CST(MIC < 0.5 μg/mL); MDR
Current antimicrobial regimens	TGC and CST	TGC, CST, meropenem and vancomycin	Cefoperazone-sulbactam, TGC	TGC, CST, and amikacin
IV/CVI/IVT tigecycline	IV,50 mg/q12h	IV, 100 mg/q12 h IVT, 2 mg/(q24h-q12h)	IV, 100 mg/q12 h IVT, (3–4)mg/q12h	IV,50 mg/q12h
Co-administered antibiotics	CST IV, 2 million IU/q8h IVT, 0.2 million IU/q24h	CST IVT, 120,000/q12h Meropenem IV, 2 g/q8h Vancomycin IV, 1 g/q12h	Cefoperazone-sulbactam IV, 3g/q12h	CST IV, 2 million IU/q6h ITH, 150,000 IU/q24h
Days to CSF sterilization	7	75	14	20
Toxicity	Renal dysfunction (CST)	Chemical ventriculitis, Myelitis(CST)	None	None
Infection outcome	Cured	Cured	Cured	Cured
Survival	NR^b^	Yes	Yes	Yes

CSF, cerebrospinal fluid; MIC, minimum inhibitory concentration; EVD, external ventricular drain; MDR, multidrug resistant; XDR, extensively drug-resistant; TGC, tigecycline; CST, colistin; q6h, every 6 h; q8h, every 8 h; q12h, every 12 h; q24h, every 24 h; IV, intravenous; CVI, continuous ventricular irrigation; IVT, intraventricular; NR, not reported;

a *On day 18 after endoscopic transsphenoidal surgery for the removal of a giant pituitary adenoma*.

b *Due to progressive worsening of renal function and GCS, the family members of the patient decided to withdraw the support due to anticipated poor neurological outcome despite microbiological cure of ventriculitis. The patient most likely died after discharge*.

## Case presentation

**Figure 1:**
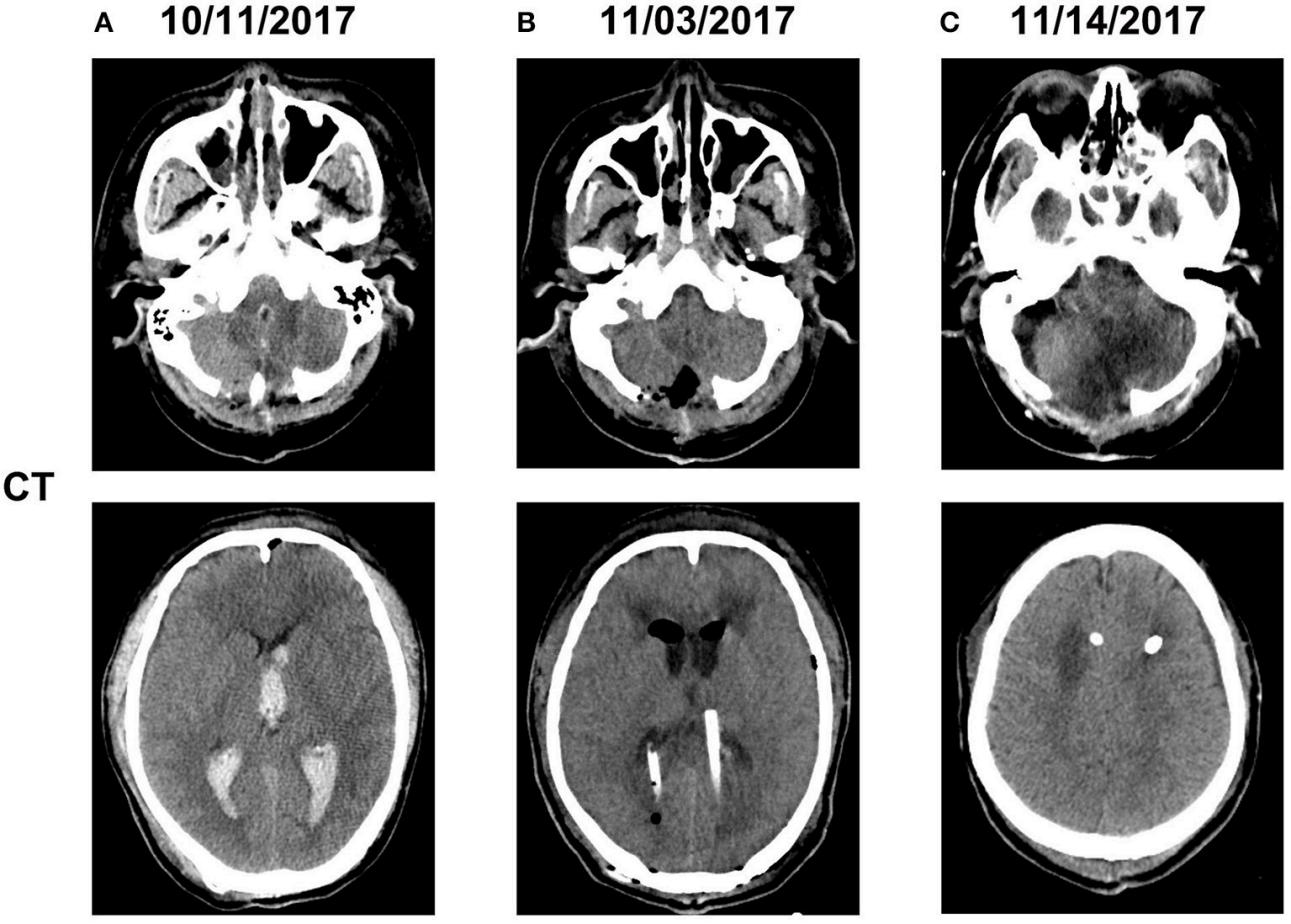
Computed tomography imaging before and after admission **(A)**. The patient underwent hematoma removal from the cerebellum and decompression in a local medical facility. Computed tomography after admission to our hospital revealed cerebellar hemorrhage in the bilateral and 3rd ventricles **(B)**. We temporarily placed a bilateral ventricular drainage in the occipital horns after the clearance of abscess on a ventriculoscope on November 3, 2017 **(C)**. We replaced the bilateral ventricular drainage in the frontal horns due to CSF leakage in the occipital horns on November 14, 2017.

A 55-year-old male patient, who was admitted to the local medical facility due to the sudden onset of severe headache and loss of consciousness, had a cerebellar hematoma on CT scan. He underwent an emergency placement of an external ventricular drain (EVD) on October 7, 2017. The patient was transferred to our hospital 3 days later because of high fever with Glasgow Coma Scale (GCS) score of 4. Follow-up CT indicated cerebellar hemorrhage in the bilateral and 3rd ventricles (Figure 1). CSF from EVD revealed a white blood cell count of 1,280 × 10^6^/L, total protein 4.18 g/L, and glucose 2.26 mmol/L, the simultaneous blood glucose level was 7.5 mmol/L (Table 3). The sputum culture tested positive for extreme-drug (EXD)-resistant *A. baumannii*, which was sensitive to amikacin only. Thus, pulmonary and intracranial infections were highly suspected, and vancomycin (1 g/day twice daily via IV) and amikacin (0.4 g/day twice daily via IV) were initiated empirically (October 12, 2017). During the follow-up period, the patient's fever gradually subsided. The laboratory CSF analysis improved, and the CSF cultures tested negative. However, the bacterial load further increased and the fever recurred.

**Table 3:** Laboratory tests for CSF in the period of treatment.

**Date**	**Oct. 13th**	**Oct. 16th**	**Oct. 19th**	**Oct. 27th**	**Nov. 06th**	**Nov. 15th**	**Nov. 18th**	**Nov. 21th**	**Nov. 24th**	**Nov. 25th**	**Nov. 28th**	**Jan. 17th**	**Normal values**
Total cells (× 10^∧^6/L)	4+/HP	25,000	9,280	310	750	30,500	2,560	3,410	180	121	30	4	< 10
WBC (× 10^∧^6/L)	1,280	320	1,090	46	75	300	0	2	2	4	0	0	< 8
Poly-karyocyte (× 10^∧^6/L)	896	288	981	37	60	270	0	0	0	0	0	0	< 5
Protein (g/L)	4.18	3.93	3.62	2.81	1.42	1.46	0.64	0.32	1.74	3.45	2.69	2.44	0.15–0.45
Glucose (mmol/L)	2.26	1.37	3.93	5.24	1.51	5.46	2.81	3.27	8.90	5.40	5.46	2.87	2.50–4.40
Chlorine (mmol/L)	120.6	127.3	116.1	113.3	127.7	124.3	136.9	138.3	118.4	126.5	124	115.8	120.0–130.0

*CSF, cerebrospinal fluid; Oct, October; Nov, November; Jan, January; WBC, white blood cell*.

**Figure 2:**
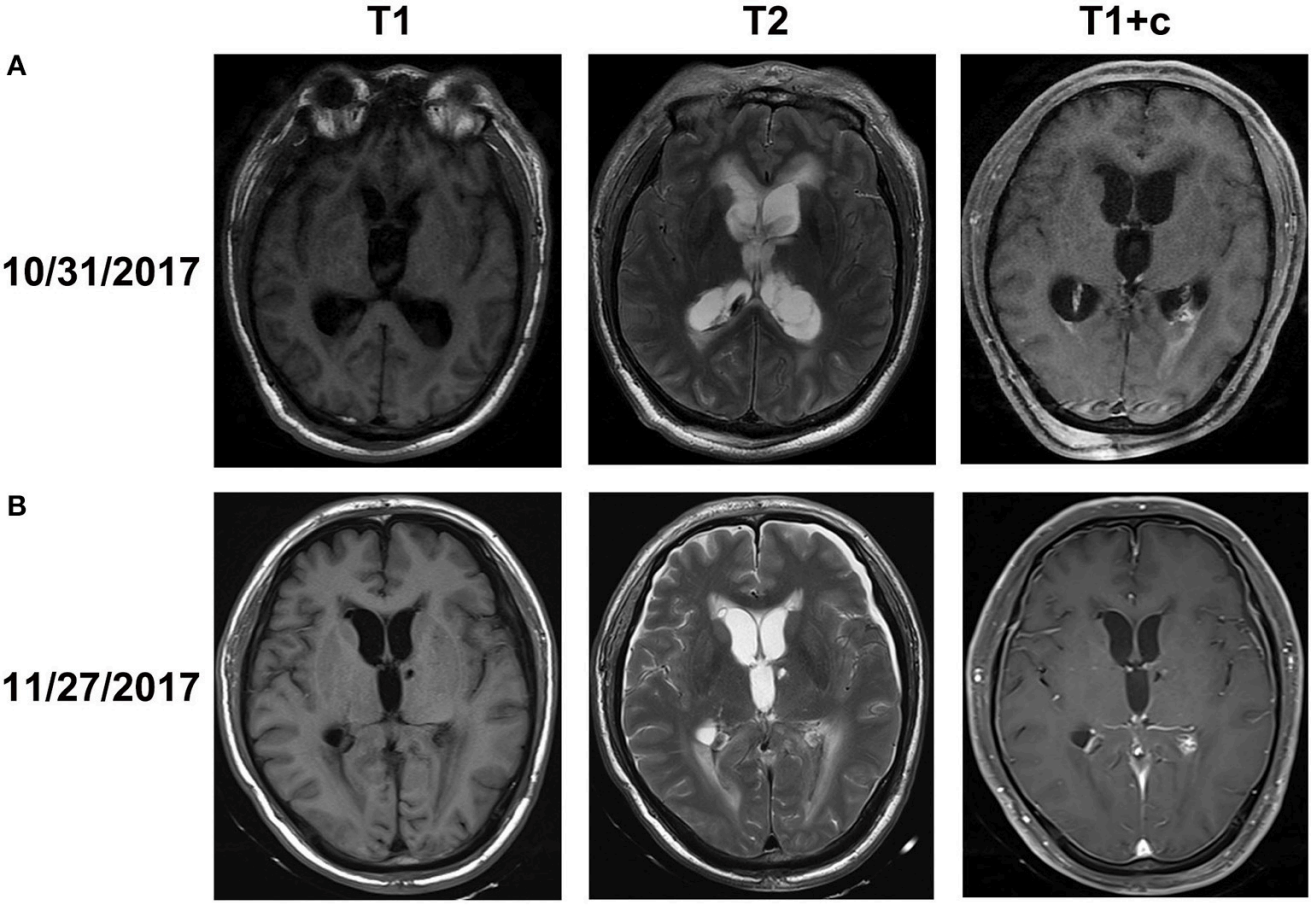
Brain MRI obtained before **(A)** and after **(B)** abscess clearance combined with tigecycline treatment **(A)**. MRI on October 31, 2017 showed that interstitial cerebral edemas beside the ventricles and bilateral ventricular occipital horn enhancement along with hydrocephalus, which indicated signs of ventriculitis **(B)**. MRI after abscess clearance combined with tigecycline treatment revealed the disappearance of the edemas, ventricular enhancement, and hydrocephalus.

**Figure 3:**
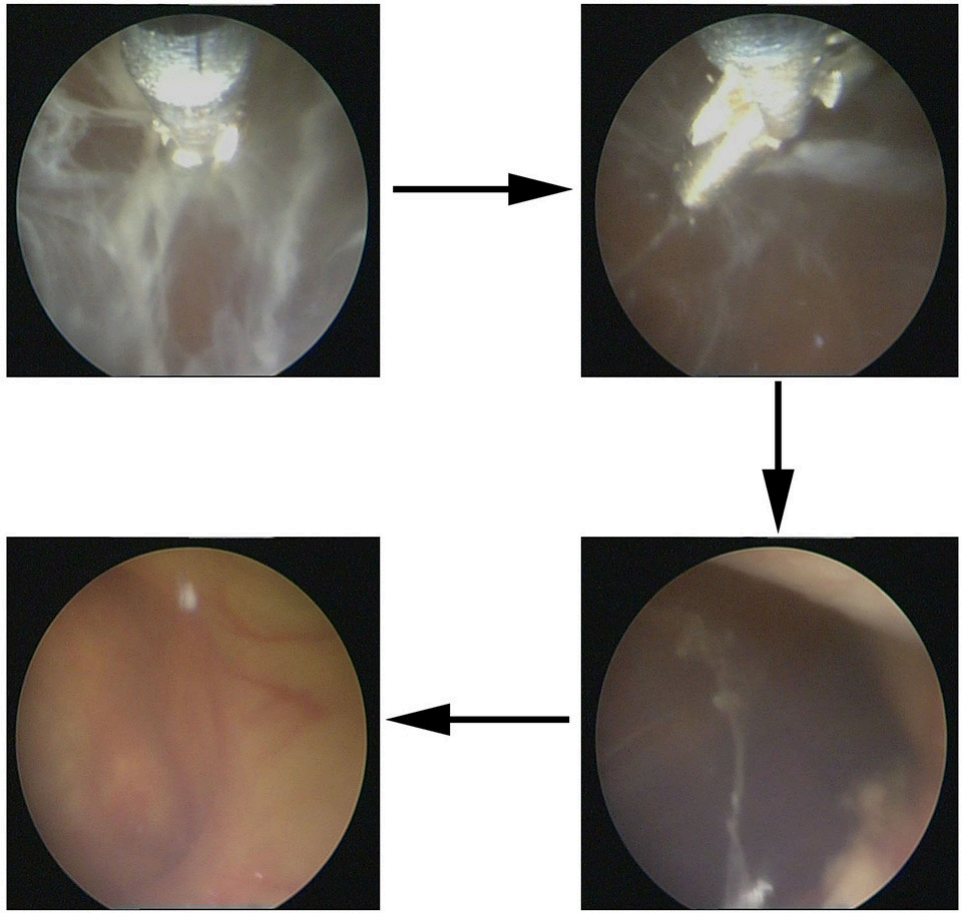
Abscess clearance on a ventriculoscope. Large amount of cellulose deposition in the ventricles were observed on a ventriculoscope, and the lesion was infected, which needed neurosurgery.

On October 31, 2017, magnetic resonance imaging revealed hydrocephalus and interstitial edema beside bilateral ventricles. In addition, occipital horn enhancement bilaterally suggested intracranial infection (Figure 2). An endoscopic ventriculostomy was done, a large amount of pus was removed from the ventricles (Figure 3), and two EVDs were placed for antibiotic therapy (bilateral occipital horn; Figure 1). The CSF cultures revealed an XDR strain of *A. baumannii* (November 6, 2017) and a minimum inhibitory tigecycline concentration of 16 μg/mL. On November 8, 2017, the patient was administered tigecycline (100 mg twice daily via IV), cefoperazone sulbactam (2 g every time, thrice daily via IV), and CVI tigecycline (10 mg/500 mL saline twice daily, in from the right occipital horn and out from the left horn). A leakage was observed around the drainage tube 4 days later. This was probably because we had to exchange the in-out tube, due to the frequent obstruction of the drainage tube or to the rather short (< 3 cm) subcutaneous tunnel of the drainage tube. Although the dressing was changed twice daily and the wound was sutured several times, the leakage persisted. Therefore, on November 14, 2017, we removed the two occipital horn drainage tubes and replaced it with two frontal horn drainage tubes with a bigger size (12#) and a longer subcutaneous tunneling (10 cm) (Figure 1). Perioperative antibiotics were administered and the leakage stopped. Twelve days from the start of the CVI tigecycline, the signs of ventriculitis disappeared, and the *Acinetobacter* CSF load steadily decreased until CSF sterilization. Thus, we adjusted the IVT tigecycline dosage to 50 mg twice daily, whereas CVI tigecycline was adjusted to IVT tigecycline (2 mg twice daily). Tigecycline was diluted in saline up to 4 mL and was slowly injected into the lateral ventricles via EVD. After every injection, the CSF drain was temporarily closed for 2 h to prevent the untimely washout of the drug. The tigecycline treatment was well tolerated by the patient. The ventricular drainage tubes were removed, and the antibiotic therapy was discontinued after 1 week of persistent negative CSF cultures and laboratory CSF analysis (Table 3). The follow-up MRI (November 27, 2017) was negative for ventriculitis (Figure 2). Thus, on November 29, 2017, 14 days after the restart of the CVI tigecycline and 3 days after the IVT tigecycline, the patient was transferred to a rehabilitation unit.

The GCS score of the patient was 10 (E4VM5) after 2 months of follow-up, and the symptoms of intracranial infection were not observed. Repeated CSF cultures were all negative, and no radiological signs of neuro-inflammation were found. The laboratory tests for CSF during follow-up (January 17, 2018) are shown in Table 3.

## Discussion

In the present report, we presented a patient with ventricular infection from XDR *A. baumannii* and they were successfully treated using abscess clearance on a ventriculoscope in combination with CVI and IVT tigecycline therapy. To the best of our knowledge, the use of such a therapeutic approach has not been previously reported. Nowadays, the IVT administration of colistin is practiced in life threatening CNS infections for *A. baumannii* meningitis/ventriculitis (12). Nevertheless, the serious nephrological and neurological side effects, such as chemical ventriculitis, chemical meningitis, and seizures, limit wide application (2, 13).

Tigecycline is a novel class of antibiotics designed to overcome drug resistance (14). It has been previously reported that tigecycline has an effect against vancomycin-resistant *Enterococci*, methicillin-resistant *Staphylococcus aureus*, penicillin-resistant *Streptococcus pneumoniae*, and numerous species of the MDR gram-negative bacteria (14). In addition, tigecycline has a favorable toxicity profile, particularly regarding adverse events involving the nervous system (14). In contrast to these advantages, the BBB penetration rate of tigecycline is only approximately 11% (14). Therefore, to limit the influence of this shortage, we considered combining IV and CVI with IVT tigecycline that may be a potential treatment alternative for our patient. CVI tigecycline in addition to IV and (or) IVT tigecycline was not used in previous studies, and yet, patients in previous studies were cured (6–11). We administered CVI tigecycline because our patients developed a local infection of the lesions in the bilateral ventricles, and a large amount of cellulose deposite was observed in the ventricles using a ventriculoscope. Moreover, we hypothesized that this new treatment, which completely cleared the abscess, is more efficient in controlling infection and reducing postoperative adhesion. Based on our results, this route was well tolerated and had excellent outcomes.

The dosages as well as the length of IV and IVT tigecycline treatment in the reported cases varied significantly. The most commonly used IV dosage ranged from 100 to 200 mg/day, and two doses were administered. However, the IVT dosage had a distribution of 2–8 mg/day. The length of intraventricular therapy also varies between 7 and 75 days in published cases (6, 7). We selected the lowest effective dosage of IV tigecycline (200 mg/day), because our patients also developed pneumonia due to XDR *A. baumannii*. With regard to IVT dosage, we chose 4 mg/day to minimize adverse effects. Twelve days after restarting CVI tigecycline, the CSF cultures tested negative, and to the best of our knowledge, this is the most rapid therapy in decreasing bacterial load without serious complications. As for the reasons why patients became infected with *A. baumannii*, based on our results and previous studies, foreign bodies, like external ventricular drain during neurosurgery and long-term neurosurgical intensive care unit (NICU) hospitalization were responsibile for those infections. We believe that this three-step approach (abscess clearance to CVI to IVT) could be important in managing MDR ventricular infections. Our patient was cured, which is similar to the previously reported cases. However, because this report only involved one patient and the use of this treatment is limited, we cannot predict the accuracy rate of the treatment and its side effects. Therefore, more studies must be conducted to demonstrate the therapeutic effects of this schedule. If confirmed to be safe and effective in the future, besides IVT colistin, this three-step approach, namely, abscess clearance in combination with CVI (bilateral frontal horn, subcutaneous tunnel >10 cm) and IVT tigecycline, could also be considered as the first-line therapy for patients with MDR intracranial infections.

## Conclusions

The use of multi-route (CVI and IVT) tigecycline and IVT colistin for MDR/XDR ventriculitis is effective, and those treatment options should be considered as a valuable therapy in managing these life-threatening intraventricular infections. However, more studies must be conducted to demonstrate the therapeutic effects.

## Ethics statement

All surgical procedures in the patient were approved by the ethics committee of Xiangya Hospital and his family members and provided written consent for publication of this case report.

## Author contributions

QL, WL, JY, JingL, JinfL, CW, XC, GP, CZ, XW, and MW: performed the surgical procedures and data analysis; WL, QL, and WZ: wrote the manuscript; QL and WZ: provided the supervision of the entire work; QL, WL, WZ, JY, JingL, JinfL, CW, XC, GP, CZ, XW, and MW: final approval of the version to be published.

### Conflict of interest statement

The authors declare that the research was conducted in the absence of any commercial or financial relationships that could be construed as a potential conflict of interest. The reviewer KD and handling Editor declared their shared affiliation.

## Abbreviations

BBB: blood–brain barrier
CSF: cerebrospinal fluid
CVI: continuous ventricular irrigation
EVD: external ventricular drain
IV: intravenous
IVT: intraventricular
MDR: multidrug-resistant
XDR: extreme drug-resistant.
